# Symptom trajectories into eating disorders: A systematic review of longitudinal, nonclinical studies in children/adolescents

**DOI:** 10.1192/j.eurpsy.2020.55

**Published:** 2020-05-26

**Authors:** Jessica McClelland, Lauren Robinson, Rachel Potterton, Victoria Mountford, Ulrike Schmidt

**Affiliations:** 1 South London and Maudsley NHS Foundation Trust, London, United Kingdom; 2 Institute of Psychiatry, Psychology and Neuroscience, King’s College London, London, United Kingdom; 3 Maudsley Health, Abu Dhabi, United Arab Emirates

**Keywords:** Child and adolescent psychiatry, comorbidity, early intervention, eating disorder, psychiatric symptoms

## Abstract

**Background.:**

Eating disorders (EDs) are serious mental illnesses that can be life-threatening. Stage of illness models and early intervention strategies could be informed by a better understanding of symptomatology that precedes the onset of an ED. This review aims to explore which symptoms (both ED and other psychiatric disorder-related) exist prior to the onset of an ED and whether there any prospective associations between these symptomatologies.

**Methods.:**

A systematic literature review was conducted in MEDLINE, Embase, and PsycINFO for large, longitudinal, prospective studies in nonclinical cohorts of children/adolescents that report symptoms prior to the onset of an ED. A quality assessment of included studies was conducted using the Newcastle-Ottawa Quality Assessment Scale.

**Results.:**

A total of 22 studies were included, and over half were assessed to be of good quality. Studies identified the presence of a broad range of ED and other psychiatric disorder-related symptoms prior to ED onset. Possible prospective associations were identified, including early eating and feeding difficulties in childhood, to ED-related symptoms (e.g., dieting and body dissatisfaction) and other psychiatric disorder-related symptoms (e.g., anxiety and depression) in childhood/early adolescence, progressing to severe symptomatology (e.g., extreme weight control behaviors and self-harm) in mid-adolescence/emerging adulthood.

**Conclusion.:**

The trajectory of symptoms identified to precede and possibly predict onset of an ED may inform early intervention strategies within the community. Suggestions for further research are provided to establish these findings and the clinical implications of these discussed, in order to inform how best to target prodromal stages of EDs.

## Introduction

The eating disorders (EDs) anorexia nervosa (AN), bulimia nervosa (BN), and binge eating disorder (BED) are characterized by aberrant eating patterns, significant psychopathology, distress, and/or impairment [1]. While disordered eating behaviors and cognitions have been reported in up to 48% of adolescents of either sex and up to 64% of female adolescents [2], full syndromal EDs affect around 8% of women and 2% of men in their lifetime. These disorders are associated with a range of physical [3] and mental health comorbidities [4,5], elevated mortality rates [6], and are difficult to treat [7]. Furthermore, EDs typically arise in early/mid-adolescence, last around 5–8 years [8,9], and are thought to be increasing in prevalence [10]. This means that they persist across the transition between adolescence and early adulthood [11], a crucial period of biopsychosocial development. As such, understanding the trajectory of symptoms leading to the onset of an ED is critical for the development of effective prevention and early intervention strategies.

Prevention and early intervention strategies are particularly pertinent given suggestions that neuroprogression may make symptoms more entrenched and less amenable to intervention over time [12]. A large body of research has evaluated preventative approaches for EDs, and selective prevention programs (i.e., those that target high-risk individuals/groups) have been shown to be most effective in reducing a range of psychopathology (e.g., body dissatisfaction, negative affect, and self-esteem[13,14]). These preventative approaches are informed by risk factor-related research. By definition, a risk factor is a measurable characteristic that precedes and increases the probability of the development of the outcome of interest [15]. In their seminal review, Jacobi et al. [16] identified a number of putative biological (e.g., gender and ethnicity), behavioral (e.g., early eating difficulties and dieting), psychological (e.g., body image difficulties and negative affect), and social (e.g., adverse experiences such as sexual abuse/physical neglect) risk factors for EDs. Recent research has added to these findings by exploring risk factors for EDs in relation to the more encompassing Diagnostic and Statistical Manual of Mental Disorders, Fifth Edition (DSM-5) criteria and found that self-objectification was the largest contributor to ED onset in young women [17]. Recently, there has also been increased interest in and evidence for genetic risk factors of EDs, which has led to the identification of specific loci for AN [18].

While many recognized risk factors for EDs may be useful in identifying, targeting, and possibly preventing EDs in those at risk of developing an ED, some of these features could also be considered as representing the early stages of—or progression into—an ED. Indeed, an at-risk state for EDs has been distinguished from a defined prodromal stage, the latter differentiated by the presence of symptoms that are features of the full disorder and indicate ED onset [12,19]. Therefore, behavioral and psychological ED risk factors (e.g., dieting and body dissatisfaction) that exist prior to ED onset may constitute ED prodromes, while biological, genetic, and environmental risk-factors (e.g., sex, early puberty, familial history, maternal factors, and adverse events) merely render an individual more susceptible to developing an ED.

An enhanced understanding of ED prodromes could inform stage of illness models of EDs and further improve emerging early intervention strategies which have shown important clinical outcomes (i.e., quick and lasting weight restoration in AN [20,21]). Indeed, the utility of exploring, defining, and treating prodromal stages of illness has been demonstrated across other psychiatric disorders [22–24]. In particular within the field of psychosis, delineation of prodromal stages [25–31] has led to interventions and services specific to this early illness phase that demonstrate significant clinical and economic impact [29,32–36]. Unfortunately, little analogous research has been conducted in relation to EDs, and very few cross-sectional, retrospective studies exist.

Given such limited research, an improved understanding of symptomatology prior to the onset of an ED that may constitute a prodromal phase is warranted. Longitudinal research in nonclinical populations of children/adolescents reporting symptomatology, as delineated in the DSM, prior to the onset of EDs could provide such initial insights. While there are many relevant existing studies, there has yet to be a systematic review of this literature or a consideration of how these findings may inform our understanding of early, prodromal stages of illness in EDs.

The current study aims to explore symptom development prior to the onset of EDs. Specifically, this review aims to address the following two questions in relation to nonclinical, adolescent populations: Which psychiatric disorder-related symptomatology exists prior to the onset of an ED? and are there any prospective associations between these symptoms?

## Methods

### Search strategy

A systematic review was conducted following the recommendations outlined in the PRISMA guidance [37]. The protocol for this systematic review was registered on PROSPERO (May 01, 2018; registration number: CRD42018094441) and is available on the PROSPERO website (http://www.crd.york.ac.uk/PROSPERO/display_record.php?ID=CRD42018094441).

Relevant studies were identified using three electronic databases (MEDLINE, Embase, and PsycINFO) and searched (via OvidSP) from inception until the date search carried out (March 05, 2020). Following are the key search terms: (eating disorder* OR anorexi* OR bulimi* OR binge eating; *title/abstract*) AND (cohort* OR longitudinal OR prospective; *title*/*abstract*) AND (symptom* OR behavio*; *title/abstract*)*.*

**Figure 1.**
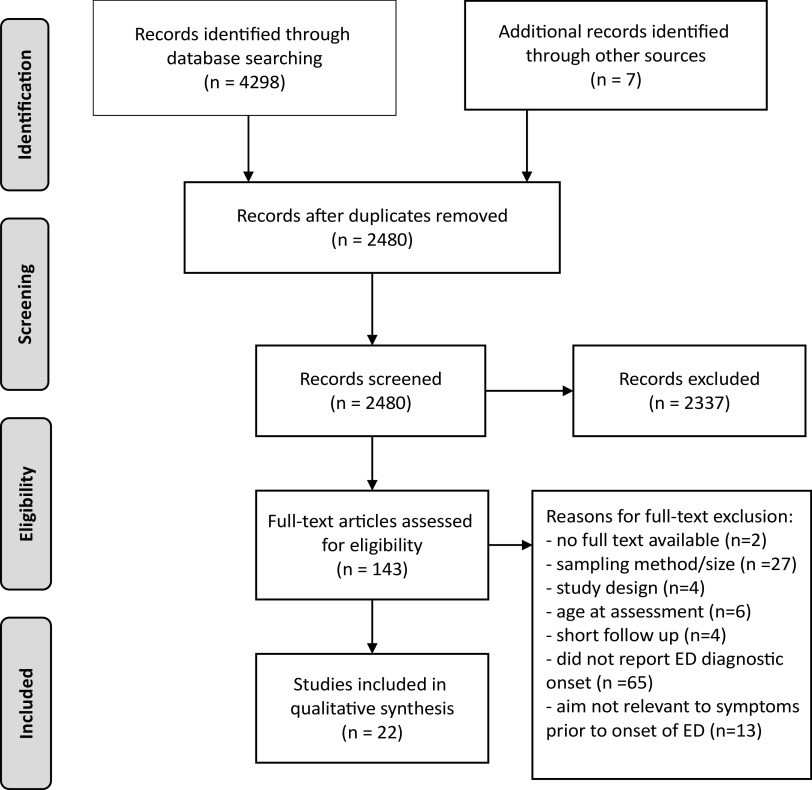
PRISMA flow chart of literature search and reviewed studies.

These searches were supplemented with Internet and hand-searches of reference lists. All identified articles were screened and included based on relevance to the topic via inspection of their title and abstract. The full text versions of the remaining articles were then assessed in more detail. An overview of the literature search is shown in Figure 1. Reviewers (J.M., L.R., and R.P.) conducted each stage of the literature search independently, and any disagreements were resolved by further examination, discussion, and via consensus.

### Inclusion criteria

We included articles in English, dated from the earliest date available up until the date the search was carried out (March 05, 2020) that aimed to explore the course/development of ED and other psychiatric disorder-related symptoms prior to the onset of an ED diagnosis including AN, BN, BED, and eating disorder not otherwise specified (EDNOS), and other specified feeding and eating disorders (OSFED). To be included in the review, studies were required to be large (*N* ≥ 100), nonclinical, population-based samples of children/adolescents (i.e., baseline assessments at 17 years of age or younger) who were followed prospectively for at least 12 months and which assessed onset of subclinical and full ED diagnoses (DSM or International Classification of Diseases-based criteria). Studies which investigated ED symptoms, without clinical or subclinical diagnoses, were not included in this review. Only studies to investigate the relation between symptoms of EDs and other psychiatric disorders, as listed in DSM and future onset of clinical or subclinical EDs, were included in this review. Behavioral and psychological ED risk factors (e.g., dieting and body dissatisfaction) that exist prior to ED onset thus have remained within the remit of this review.

Reviewers (J.M., L.R., and R.P.) extracted data from studies regarding sample characteristics, study design, and relevant findings. These reviewers also assessed the quality of each study, with discrepancies discussed and agreed upon following consultation with experts from the research group. The quality of included studies was appraised using the Newcastle-Ottawa Quality Assessment Scale (NOS) for cohort studies [38]. This scale was developed to assess the quality of nonrandomized studies in systematic reviews and meta-analyses. The NOS judges study quality on eight items from three broad criteria—the selection of the study groups, comparability of the groups, and the ascertainment of the outcome of interest. A maximum score of 9 can be given to a study and thresholds for converting the NOS to Agency for Healthcare Research and Quality [39] standards are as follows—“Poor”: selection ≤ 1 OR comparability = 0 OR outcome/exposure ≤ 1; “Fair”: selection = 2 AND comparability ≤ 2 AND outcome/exposure ≤ 3; “Good”: selection ≤ 4 AND comparability ≤ 2 AND outcome/exposure ≤ 3.

## Results

### Characteristics of included studies

We identified 22 studies (see Table 1) that met inclusion criteria for this review, and percentage agreement between reviewers for inclusion was 100%. These 22 studies included 56,164 participants from 15 cohorts across 6 countries. On average, individuals were 12 years of age at initial assessment (exclusion of two birth cohorts that did not report age at assessment), 49% male (aside from one female only study), and were followed up for a period of 8 years (minimum = 1.5 years; maximum = 30 years). In terms of the age at which studies assessed symptoms prior to ED onset, 2 out of 22 studies followed individuals from birth, 4 out of 22 began during childhood, and most studies (16/22) followed adolescents (i.e., between 12 and 17 years of age). Studies determined and reported ED onset through self-report (8/22), validated questionnaires (8/22), and/or interview measures (7/22) based on DSM-III-R, DSM-IV, or DSM-5 diagnostic criteria. A combination of full and partial/subthreshold ED diagnoses were investigated—most studies investigated full threshold ED cases (14/22), with others investigating a combination between full and subthreshold/partial EDs (8/22).

**Table 1.** Characteristics and findings of reviewed studies.

References	Country	*N*	Cohort	Age (*M* ± SD)	Gender	Follow-up	ED diagnosis	ED diagnostic threshold	Findings	Quality
Ackard et al. [40]	United States	2,516	Project EAT-II; 31 schools	20.4 **±** 0.8 at follow-up	45% male	5 years	Self-report questions re: ED criteria (DSM-IV)	Full threshold ED cases	2.2% males and females with body image disturbance developed BED or BN. 0.9% of males and 3.7% of females with binge eating or compensatory behaviors developed BED, or BED and BN, respectively.	Poor
Allen et al. [41]	Australia	1,597	Raine study; birth cohort	14.1 **±** 0.1 at follow-up	55% male	14 years	Adapted child EDE and EDEQ (DSM-IV)	Full, partial, and “at risk” ED cases	A restricted diet predicted later ED cases.	Good
Beato-Fernandez et al. [42]	Spain	1,076	22 schools	12–13 at baseline	46% male	2 years	EAT-40 (DSM-III-R/IV)	Full (AN, BN) and EDNOS cases	Body dissatisfaction predicted later ED diagnosis. Those who developed an ED had more suicidal and self-harm tendencies, worse depressive symptoms, and body dissatisfaction at baseline.	Poor
Herle et al. [43]	United Kingdom	4,760	ALSPAC	1.3 at baseline	52% male	8.7 years	Data from YRBSSQ and DEBQ (DSM-5)	Full threshold ED cases	Childhood overeating, persistent undereating, and persistent fussy eating were predictive of adolescent binge eating disorder, anorexia nervosa (girls only), and anorexia nervosa (whole sample), respectively.	Good
Johnson et al. [44]	United States	726	Families from two NY counties	13.8 **±** 2.6 at baseline	49% male	10 years	DISC-I (DSM-III-R)	Full Threshold ED cases and ED behaviors	Depressive and anxiety disorders were predictive of EDs. Disruptive and substance use disorders were not predictive of EDs.	Good
Killen et al. [45]	United States	887	4 schools	12.4 **±** 0.7 at baseline	All female	3 years	Self-report questions (diagnostic tool not reported)	Full and partial ED cases	Elevated weight concerns associated with onset of partial EDs. Earlier drive for thinness, body dissatisfaction, perfectionism, and restraint most characteristic of those who later develop partial EDs	Poor
Killen et al. [46]	United States	825	4 schools	14.9 at baseline	All female	4 years	Interview and adapted EDE (DSM-III-R)	Full and partial ED cases	Elevated weight concerns associated with onset of partial EDs. Earlier drive for thinness, bulimia, body dissatisfaction, ineffectiveness, interoceptive awareness, temperament (distress, fear), dieting, restraint, and alcohol use most characteristic of those who later develop partial EDs.	Good
Kotler et al. [47]	United States	976	Families from two NY counties	6.1 at baseline	50% male	17 years	DISC (DSM-III-R/IV)	Full threshold ED cases and ED behaviors	Eating conflicts, struggles with food and unpleasant meals predicted AN.	Good
Liechty and Lee [48]	United States	14,322	Add Health; 80 high schools	15.9 **±** 1.8 at baseline	49% male	7 years	Self-report (ever told have an ED? yes/no)	Implied full threshold ED cases	Depression, body image dissatisfaction (males only), and extreme weight loss behaviors (females only) was associated with unspecified ED diagnosis.	Good
Marchi and Cohen [49]	United States	659	9 schools	~6 years at baseline	51% male	10 years	DISC (DSM-III-R)	Full threshold ED cases	Risks in early childhood for subsequent symptoms of anorexia nervosa include picky eating and digestive problems. Early symptoms of AN/BN predictive of later diagnosis and pica predictive of BN.	Good
Neumark-Sztainer et al. [50]	United States	2,516	Project EAT-II; 31 schools	12.8/15.8 **±** 0.8 at baseline	45% male	5 years	Self-report (ever told have an ED? yes/no)	Implied full threshold ED cases and ED behaviors	In females, dieting significantly associated with later EDs.	Poor
Nicholls and Viner [51]	United Kingdom	16,567	BCS70; birth cohort	From birth	56% male	30 years	Self-report (ever told have an ED? yes/no)	Implied full threshold ED cases	Infant feeding problems, under eating, and increased exercise (at 10 years) associated with later AN.	Poor
Nicholls et al. [52]	United Kingdom	16,567	BSC70; birth cohort	From birth	49% male	30 years	Self-report (ever told have an ED? yes/no)	Implied full threshold ED cases	Eating, sleep problems, and overeating (5 years) associated with later BN/BED.	Poor
Patton et al. [53]	Australia	1,947	45 schools	14.5 **±** 0.5 at baseline	47% male	3 years	BET (DSM-IV)	Full and partial syndrome ED cases	In females, severe and moderate dieting increased risk of future ED. Psychiatric morbidity (independent of dieting) predicted onset of EDs.	Poor
Penas-Lledo et al. [54]	Sweden	615	TChAD; Twin study	16–17 years at baseline	All female	3 years	Self-report (ever had BN or AN? Yes/no)	Implied full threshold ED cases	Drive for thinness predicted later BN. Interaction between drive for thinness and anxious/depressed mood predicted risk of both BN and AN.	Poor
Ranta et al. [55]	Finland	3,278	Finnish Adolescent Mental Health Cohort Study	15.5 **±** 0.4 at baseline	51% male	2 years	Self-report questions re: ED criteria (DSM-IV-TR)	Implied full threshold ED cases	Depression predicted AN, while social phobia and depression both predicted BN. However, controlling for initial comorbidity, Eds, and socioeconomic factors removed this effect.	Poor
Schaumberg et al. [56]	United Kingdom	7,767	ALSPAC	10 years	Not reported	6 years	Data from YRBSSQ & DEBQ (DSM-5)	Full threshold ED cases	Physical anxiety symptoms predicted BN, while worries (e.g., about the future) predicted AN.	Fair
Stice et al. [57]	United States	496	8 schools	13.5 **±** 0.7 at baseline	All female	2 years	EDE (DSM-IV)	Full and partial threshold ED cases	Depressive symptoms (but not substance abuse) predicted onset of subthreshold BN.	Good
Stice et al. [58]	United States	496	8 schools	15.4 **±** 0.7 at baseline	All female	5 years	EDDI (DSM-IV)	Full and partial threshold ED cases	Fasting (not eating for 24 hours) for weight control more predictive of future sub/full BN than dietary restraint.	Good
Stice et al. [59]	United States	496	8 schools	13.5 **±** 0.7 at baseline	All female	8 years	EDDI (DSM-IV)	Full and partial threshold ED cases	Body dissatisfaction predictive of ED onset. Those with high or low body dissatisfaction and elevated depressive or dieting associated with increased ED onset respectively.	Good
Stice and Van Ryzin [60]	United States	496	8 schools	13.5 **±** 0.7 at baseline	All female	8 years	EDDI (DSM-IV)	Full and partial threshold ED cases	Growth curve models showed perceived pressure to be thin and/or thin-ideal internalization, before showing onset of disorder-predictive levels of body dissatisfaction, before showing onset of disorder-predictive levels of dieting and/or negative affect, before showing onset of the ED.	Good
Wilkinson et al. [61]	United Kingdom	945	Roots study	14 at baseline	47% male	3 years	K-SADS-PL (DSM-IV)	Full threshold ED cases	Recurrent nonsuicidal self-injury predicted onset of EDs.	Good

Abbreviations: ALSPAC, UK Avon Longitudinal Study of Parents and Children; AN: anorexia nervosa; BCS70, 1970 British Cohort Study; BED: binge eating disorder; BET, Branched Eating Disorders Test; BN: bulimia nervosa; DEBQ, Dutch Eating Behavior Questionnaire; DISC-II, Diagnostic Interview Schedule for Children, Version 2; DSM (-III-R/-IV/-5), Diagnostic and Statistical Manual of Mental Disorders, Third (Revised)/Fourth/Fifth Edition; EAT-40: Eating Attitudes Test; ED, eating disorder; EDDI: Eating Disorder Diagnostic Interview; EDE, Eating Disorder Examination; EDE-Q, Eating Disorder Examination Questionnaire; EDI: Eating Disorder Inventory; EDNOS, Eating Disorder Not Otherwise Specified; KSADS-PL, Kiddie Schedule for Affective Disorders and Schizophrenia, Present and Lifetime version; Project EAT-II, Eating Among Teens; TChAD, Twin study of Child and Adolescent Development; YRBSSQ, Youth Risk Behavior Surveillance System Questionnaire.

### Quality of included studies

As detailed in the NOS, the quality of each study was assessed based on three broad categories pertaining to sample selection, comparability, and outcome evaluation. The percentage agreement between reviewers when assessing the quality of studies was 80%, with inconsistencies resolved via discussion. The quality assessment rating was *poor* for 41% (9/22), *fair* for 4% (1/22), and *good* for 55% (12/22) of studies. In terms of sample selection, 81% (18/22) of studies were found to be adequately (truly/somewhat) representative of children/adolescents in the community, all studies included individuals from the same community, 68% (15/22) used reliable methods (secure record and structured interview or questionnaire) to measure symptoms prior to ED onset, and 71% (15/22) demonstrated that EDs were not present at the start of the study (this included birth cohort studies). The proportion of studies that did not report details relating to representativeness was 18% (4/22). In terms of comparability, almost all studies (21/22) controlled for age, sex, and/or initial ED symptoms in their analyses (this included studies that were only in females or excluded individuals with EDs at the start of the study). In terms of follow-up, 59% (13/22) and 63% (14/22) of studies followed participants for at least 5 years and had adequate (>70%) follow-up rates, respectively.

### Study findings

Studies reported the presence of a broad range of ED and other psychiatric disorder-related symptoms prior to ED onset. These are discussed and summarized in Table 1.

#### ED symptoms

Several studies reported that early eating difficulties were present prior to and predicted future EDs. Marchi and Cohen [49] first reported this in 6-year-olds followed for approximately 10 years. They reported that pica in early childhood was associated with later BN, as well as early AN/BN symptoms with later diagnosis. Early eating difficulties (e.g., digestive problems and picky eating) were also associated with later AN/BN symptoms. In a comparably aged group of children followed for up to 17 years, struggles and conflicts with food, as well as unpleasant mealtimes, predicted AN [47]. Two later studies demonstrated that at 30 years follow-up, infant feeding problems and undereating predicted and preceded AN while early overeating preceded and predicted BN/BED, respectively [51,52]. Most recently, childhood overeating, persistent undereating, and persistent fussy eating were predictive of adolescent BED, AN in girls only, and AN within the whole sample, respectively [43].

Several studies reported the preceding and predictive nature of body dissatisfaction on EDs. Beato-Fernandez et al. [42] found that body dissatisfaction predicted ED diagnosis in a group including AN, BN, and EDNOS 2 years later. Interestingly, two studies found that the presence of body image disturbance or dissatisfaction on the later development of BED/BN or EDs, respectively was only relevant to males [40,48]. The interaction between drive for thinness/body dissatisfaction and other symptomatology (e.g., anxious/depressed mood and dieting) on predicting EDs such as BN and AN was observed in several studies [59,60,62]. Growth curve models showed perceived pressure to be thin, and/or thin-ideal internalization predated the onset of disorder-predictive levels of body dissatisfaction, following disorder-predictive levels of dieting and/or negative affect, before showing onset of the ED [60]. Dietary restriction and dieting to control weight were all associated with future EDs, in particular BN or BED [63] or binge-related symptoms. Of note, Liechty and Lee [48] measured binge-related symptoms using the following question: “In the past seven days, have you been afraid to start eating because you thought you would not be able to stop or control your eating?” which may not capture an objective binge episode.

Other extreme dietary/compensatory behaviors in females, for example, self-induced vomiting and laxative use, have been reported to precede EDs, in particular BED and BN [40,48]. In Project EAT-II, among the 301 girls at time 1 with body image disturbance who did not endorse any binge eating or use of compensatory behaviors, nearly 30% worsened such that they reported binge eating, the use of compensatory behaviors, or met threshold diagnostic criteria for BN or BED. Conversely, less than one-fifth of the girls with BED or BN at time 1 were asymptomatic at time 2 [40]. In addition, “extreme weight loss behaviors,” including any reporting one or more of the following: diet pills, laxatives, or vomiting within the past 7 days to lose weight, were predicted a later self-reported ED diagnosis [48].

#### Other psychiatric disorder-related symptoms

The presence of other psychiatric disorder-related symptoms prior to the onset of an ED was reported in several studies. Both Johnson et al. [44] and Liechty and Lee [48] found that in adolescents (13/15 years of age), depression was associated with EDs up to 10 years later; however, the latter study found this only in males. Other findings suggest depressive symptoms (but not substance abuse or social phobia) predicted the onset of full/sub-threshold BN within the next 2 years [55,57]. However, findings in relation to AN were not significant when controlling for baseline comorbidity, EDs, and socioeconomic factors [54,59]. As previously mentioned, two studies noted the interaction between depressive mood and body dissatisfaction/drive for thinness in predicting EDs [54,59].

Along with depressive symptoms, anxiety was also found by Johnson et al. [44] to be predictive of EDs; however, this was dependent on whether responses to anxiety and/or later ED symptoms were ascertained via individual or maternal interview. As mentioned, an interaction between anxious/depressed mood and drive for thinness was found by Peñas‐Lledó and Bulik [62], and in this study, social phobia was found to precede BN, but not AN [55], albeit not when initial comorbidity, EDs, and socioeconomic factors were accounted for. In a recent study of children followed for 6 years, symptoms of generalized anxiety, specifically physical anxiety symptoms (e.g., feeling tense) were found to precede illness onset of BN and worries (e.g., about the future) were found to precede that of AN [56].

Findings related to other psychiatric disorder-related symptomatology were reported in some studies. For example, Killen et al. [46] found that alcohol use in 15-year-old girls was characteristic of those who developed EDs 4 years later. Patton et al. [53] reported that psychiatric morbidity, independent of dieting, predicted onset of EDs. Finally, in a recent study, Wilkinson et al. [61] reported that recurrent nonsuicidal self-injury behavior in 14-year-olds was predictive of EDs 4 years later [61].

#### Findings in relation to AN

As mentioned, childhood (i.e., under 5 years of age) eating problems (e.g., eating conflicts, struggles with food, and undereating) were found to be associated with AN up to 30 years later [47,51]. In addition, persistent undereating was associated with higher AN risk in adolescent girls only, and persistent fussy eating was associated with greater AN risk in girls and boys by 16 years of age [43]. One study found that depression alone predicted AN 2 years later [55]; however, not when comorbidity, EDs at baseline and socioeconomic factors were controlled for. The interaction of drive for thinness and anxious/depressed mood was associated with AN [54] and in terms of anxiety alone, generalized worries (but not social phobia specifically) were associated with later AN [55,56].

#### Findings in relation to BN and BED

Early sleeping difficulties and overeating were found to be characteristic of those who developed BN/BED-type EDs up to 30 years later [52]. In addition, childhood overeating was predictive of future BN and BED at 16 years of age [43]. In both males and females, body image disturbance and binge eating/compensatory behaviors were associated with BED and BN, respectively [40]. The interaction of drive for thinness and anxious/depressed mood was found to be associated with BN [54]. Physical anxiety-related symptoms also predicted BN [55,56]. Social phobia and depression (but not substance abuse) predicted BN [57] as well as physical anxiety-related symptoms [55,56].

#### Prospective associations between symptoms

**Figure 2.**
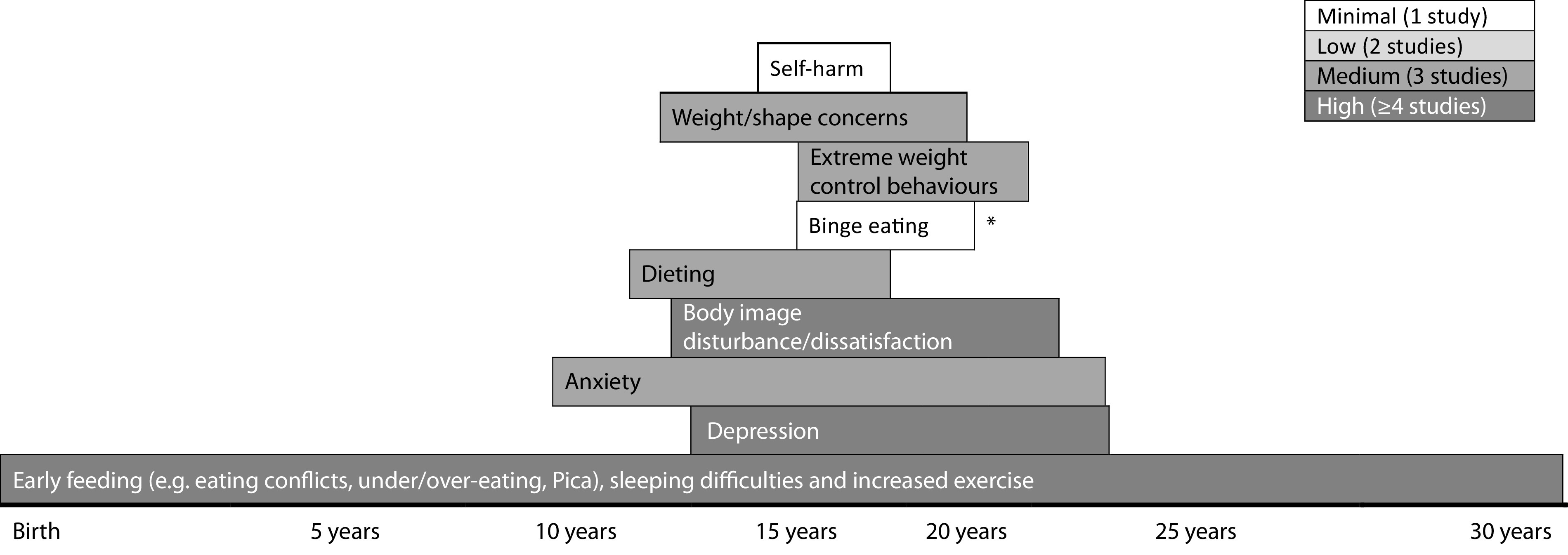
Emergence of symptoms prior to eating disorder onset. *This study was in relation to binge eating disorder only; length of rows indicates points of baseline and follow up assessments.

As summarized above and in Figure 2, the earliest symptoms reported to be associated with future EDs (up to 30 years later) are problematic eating during early childhood, that is, under 10 years of age [41,47,49,51,52]. Then, during middle childhood/early adolescence (i.e., from 10 to 13 years of age), a range of symptoms emerge, including those relating to ED, that is, dieting [45,46,50,53,58], body dissatisfaction [40,42,45,48], and weight/shape concerns [45,46,54,59]; other psychiatric disorder-related, that is, anxiety [55,56] and depression [44,48,55,57]; [64]. Following these and at around 15 years of age, more severe symptoms such as binge eating, fasting, compensatory weight loss behaviors, and nonsuicidal self-injury arise and precede ED onset during the early 20s [40,48,58,61].

## Discussion

To the best of our knowledge, this is the first systematic review of longitudinal cohort studies regarding the development of symptoms prior to the onset of diagnoses of EDs. This review aimed to address two questions: The extent to which ED symptoms and other psychiatric disorder-related symptoms occur prior to the onset of an ED [1] and the prospective relationship between these symptoms and ED diagnostic criteria including AN, BN, BED, and EDNOS or OSFED [2].

Our review highlights that the symptoms which exist prior to the onset of an ED are largely consistent with current diagnostic criteria of EDs and associated comorbidity. More specifically, our findings suggest that a range of ED symptoms (e.g., early eating difficulties, dietary restriction, fasting, body dissatisfaction, and weight/shape concerns), other psychiatric disorder-related symptoms (e.g., depression and anxiety) exist prior to the onset of EDs. Additionally, our review provides preliminary evidence that prospective associations may exist between these symptoms. Specifically, we found that eating difficulties in early childhood preceded the appearance of a cluster of ED (e.g., dieting, body dissatisfaction, and weight/shape concerns) and other psychiatric disorder-related symptoms (e.g., anxiety and depression) in middle childhood/early adolescence and later, more severe symptoms (e.g., binge eating, compensatory weight control behaviors, and self-harm) which tend to arise during mid-adolescence/emerging adulthood [47,48,60].

In terms of ED-specific symptoms prior to future illness onset, a significant proportion of studies indicate an age-related trajectory in their development. For example, childhood eating difficulties are the first to arise [43,47,49,51,52]. Interestingly, these are consistent with ED phenotypes, for example, conflicts, struggles, and undereating in relation to AN and overeating in relation to BN, both up to 30 years later [23,47,51,52]. However, larger studies and replication are required to tease apart possible ED diagnosis–specific trajectories into illness. These early eating difficulties were found to predate the emergence of late childhood/early adolescent body image dissatisfaction (particularly relevant for males and BN/BED-type EDs), weight/shape concerns, and dietary restraint [40,42,48,54], followed by the appearance of more extreme dieting/weight control behaviors (e.g., binge eating, fasting, self-induced vomiting, and laxative use) in mid/late adolescence [40,48]. These findings are similar to those reported by Allen et al. [63], in that, more established ED symptoms (e.g., purging, fasting, and binge eating) appear to persist between mid-adolescence/early adulthood. Additionally, the adolescent symptom clusters identified in this review tended to be associated with ED onset during emerging adulthood, apart from dietary restraint which often emerged at an earlier age and was associated with earlier ED onset. This is consistent with other findings whereby childhood undereating predicted the onset of adolescent AN [43]. It is important to note that while such findings could represent EDs in evolution and possible ED prodromes, these trajectories of ED symptoms could also be age-specific manifestations of full diagnoses and represent shortcomings of the current diagnostic criteria when applied to children/adolescents [65].

In terms of other psychiatric disorder-related symptoms, anxiety and depression were shown to commonly exist prior to ED onset [44,56]. This is consistent with other findings relating to the prediction of affective symptomatology on later ED symptoms [66–71]. However, studies reviewed here also suggest that affective symptomatology may appear concurrently [55] or interact with ED-related symptoms [54]. This latter finding has also been established in longitudinal studies investigating the onset of subthreshold EDs or ED symptoms, suggesting that the presence of early ED and affective symptoms (e.g., dietary restraint and depression) predict later ED behaviors/attitudes [72,73]. Fewer studies report the presence of other psychiatric disorder-related symptoms, such as alcohol use and nonsuicidal self-injury prior to EDs [46,61]. Overall, the presence of other psychiatric symptoms prior to ED onset is unsurprising given the high rates of lifetime comorbidity associated with EDs [5]. However, as the studies reviewed here and those outside the scope of this review have demonstrated, there is mixed evidence for any prospective association between affective and ED symptoms/disorders [74–76]. Further longitudinal research with frequent assessment points is warranted in order to tease apart any chronological patterns in the appearance of ED and other psychiatric disorder-related symptomatology or understand how they collectively contribute to the onset of EDs.

It is important to note that a number of important studies in this field were not included as they were outside the scope of the current review (e.g., investigated broad predictors of EDs or measured ED symptoms rather than diagnoses as outcomes). Specifically, findings that early traits such as impulsiveness and low self-esteem/efficacy are associated with EDs up to 30 years later [41,51,52] are particularly relevant for BN/BED-type EDs. Similarly, the predictive nature of hyperactivity/inattention and self-esteem on binge eating and purging behaviors [77,78] and symptom trajectories of hyperactivity and inattention subscores are predictive of later EDI subscales, including restrictive-eating and bulimia subscales [64]. In addition, there is a comprehensive literature on the relationship between EDs and autistic traits [79–82], although no studies to date have investigated autistic traits as predictors of future ED diagnoses in longitudinal cohort-based studies.

This is the first study to review longitudinal, cohort-based studies in relation to symptomatology prior to ED diagnostic onset. This systematic review involved independent double screening at all possible stages with minimal disagreement. Stringent inclusion criteria relating to sample sizes, length of follow-up, and the requirement for studies to report the progression to an ED were adhered to. Additionally, a thorough assessment of the quality of included studies was conducted. These factors demonstrate the originality of this review, provide confidence in its inclusion of relevant literature, and the thorough way in which this was reviewed.

There are several limitations to this review which must be considered. First, studies were heterogeneous in terms of their aims, design, methodology, and findings, making comparisons of findings difficult, meta-analyses impossible, and the generalizability of overall findings limited. Second, studies reviewed were based on stringent ED diagnostic criteria (i.e., DSM-III or DSM-IV) or self-report of an ED diagnosis from a medical professional, and therefore this review is likely to have missed information from many individuals who would meet current diagnostic criteria for an ED of subthreshold ED, given the broader consideration of symptoms (e.g., elimination of amenorrhea criterion in AN and reduction of binge/purge frequency in BN/BED) and new diagnoses (e.g., BED and avoidant/restrictive food intake disorder) in DSM-5. Third, we focused primarily on psychiatric disorder-related symptoms prior to the onset of EDs; therefore, are likely to have omitted other important factors that could constitute an ED prodrome (e.g., overweight/obesity and personality traits). Finally, it would have been informative to consider a psychiatric comparison group in order to evaluate how ED prodromes may differ from prodromal stages of other psychiatric disorders.

The identification of symptom trajectories into EDs and possible prospective associations elicited from the current review could have important clinical and research implications. For example, our findings inform stage models of illness in EDs, specifically the early prodromal phase. This improved understanding of ED prodromes could be useful in informing early intervention programs. Specifically, these could target a range of symptomatology earlier (i.e., early eating difficulties in childhood, affective symptomatology in childhood, dieting, and depression in early adolescence). Other studies have also suggested this in relation to the impact of childhood symptoms on later disordered eating [73,83]. In terms of research implications, a significant proportion of potentially relevant studies were excluded from this review as they did not report progression of symptoms into a clinical ED diagnosis. Future longitudinal research should aim to report this information. Additionally, efforts should be made to ensure large cohorts of children/adolescents are evaluated which are characteristic of the wider community, and data regarding representativeness should always be reported. Standardized, well-validated measures to assess a broad range of symptomatology prior to ED onset should be used, preferably consistently across studies. Assessment of EDs at baseline should be conducted, relevant individuals excluded, and analyses should control for important factors such as age and sex, as has been done in some of the highest quality papers included in this review [43,55]. Assessment of ED onset should preferably be via clinical interviews based on current diagnostic criteria (e.g., Eating Disorder Examination) and if not, via the use of well-validated questionnaire equivalents (e.g., Eating Disorder Examination Questionnaire). Follow-up should include early (i.e., before adolescence) and regular (i.e., yearly) assessments, over a long enough time period that would enable a better understanding of possible prospective associations of a broad range of symptomatology. Finally, larger sample sizes (ideally via combining information across similarly designed studies) and the inclusion of clinical comparison groups would help to elucidate symptom trajectories that are specific to EDs (e.g., compared to depression and body dysmorphic disorder), to individual ED diagnoses (e.g., AN compared to BN or BED), or differ according to factors such as sex.

Prospective and longitudinal designs as described would represent a gold standard in the assessment of symptoms prior to illness onset and therefore in evaluating prodromal stages of EDs. However, it is important to note that it was uncommon for a large population-based cohort study to be designed for the specific purpose of measuring trajectories into illness in ED diagnoses. Instead, many were designed to present the research community with a rich dataset to understand genetic and environmental influences on health and development, which were then used by researchers to investigate the trajectories into ED diagnoses. While retrospective assessments of symptoms prior to an ED are limited by biased retrospective recall or subjective, inaccurate memory, they have been shown to be informative in other psychiatric disorders such as psychosis [84]. Retrospective assessments could be equally informative in advancing our understanding of prodromal stages of EDs.

In conclusion, this review identified the presence of a broad range of psychiatric symptomatology that precedes and may be indicative of future ED onset, as well as an insight into potential prospective associations. These symptom profiles may represent prodromal stages of EDs; however, further work is required to establish these findings. Such research could inform early intervention strategies and ultimately deter the progression of EDs into chronic, life-threatening conditions.

## Data Availability

As this is a systematic review, there are no empirical data. Qualitative resources (e.g., spreadsheets of studies evaluated) are available on request from the authors.
